# Developmental outcomes and physical activity behaviour in children post major surgery: an observational study

**DOI:** 10.1186/s12887-016-0660-4

**Published:** 2016-08-03

**Authors:** Genevieve Mary Dwyer, Karen Walker, Louise Baur, Nadia Badawi

**Affiliations:** 1 Physiotherapy Program, School of Science and Health, The University of Western Sydney, Locked Bag 1797, Penrith, NSW 2751 Sydney, Australia; 2 Grace Centre for Newborn Care, The Children’s Hospital at Westmead, Sydney, Australia; 3 Discipline of Paediatrics and Child Health, Sydney Medical School, The University of Sydney, Sydney, Australia; 4 Cerebral Palsy Alliance Research Institute, The University of Sydney, Sydney, Australia

**Keywords:** Infant, Child, Early childhood, Neonatal surgery, Developmental outcomes, Neurodevelopment, Cognitive, Motor, Language development, Physical activity behaviour, Sedentary behaviour, Small screen recreation, Bayley Scale of Infant and Toddler Development, Third edition, Preschool-Age Physical Activity Questionnaire, Follow-up

## Abstract

**Background:**

Infants may be at neurodevelopmental risk from adverse events arising in the neonatal period. This study aimed to investigate the developmental outcomes and physical activity behaviours of term infants after neonatal major surgery, at age three years.

**Methods:**

This prospective study enrolled infants who underwent major surgery in their first 90 days, between August 2006 and December 2008. Developmental status was assessed using the Bayley Scales of Infant and Toddler Development, Third Edition (BSID-III). Physical activity and sedentary behaviour (i.e. small screen recreation) (SSR) were assessed using the Preschool-Age Physical Activity Questionnaire (Pre-PAQ). Activity (moving between slow to fast pace) and SSR were reported for a 3-day period.

**Results:**

One hundred and thirty five children (68 major surgery, 67 control) were assessed, using both measures, at age three years. Both groups were within the average range across all domains of the BSID-III although the surgical group was significantly below the controls for cognition (*t* = −3.162, *p* = 0.002) receptive language (*t* = −3.790, *p* < 0.001) and fine motor skills (*t* = −2.153, *p* = 0.03). Mean activity time for the surgical group was 191 mins.day^−1^, and 185 mins.day-1 for controls. Mean SSR time was 77 mins.day^−1^, and 83 mins.day^−1^ for the respective groups. There was no significant difference between groups for either physical activity (*p* = 0.71) or SSR time (*p* = 0.49).

**Conclusions:**

By age three, children who had major surgery in infancy are developmentally normal but have not quite caught up with their peer group in cognitive, receptive language and fine motor skill domains. Both groups met recommended 3 h of daily physical activity but exceeded 60-min SSR time recommended for preschool-age children.

## Background

Infants may be at neurodevelopmental risk from adverse events arising in the neonatal period. These events include the consequences of prematurity or the presence of congenital anomalies, requiring surgical correction during the early period of life. While the developmental sequelae due to prematurity have been well documented for over 25 years [1–4], the developmental outcomes of term infants who have undergone major surgery in the early stage of life have not been as well investigated. Gischler et al. [5] noted survival alone is no longer a sufficient parameter for successful treatment in newborns requiring surgical intervention. The short and longer-term developmental outcomes of infants who have undergone cardiac surgery have gained increasing interest in the last decade but research is still comparatively limited despite improved survival rates [6–9]. Even less is known about the developmental outcomes of infants who have undergone non-cardiac major surgery [10–12].

The present study is part of an ongoing multicentre project undertaken to ascertain the long term outcomes of a cohort of infants who underwent cardiac or other non-cardiac major surgery in the first 90 days of life, and to compare the outcomes with a healthy born peer cohort recruited at the same time. At age one year major surgery was found to be significantly associated with developmental delay. Delay was found in all areas (cognition, receptive language, expressive language, and fine and gross motor development) but was greatest in the area of gross motor development and particularly in infants who underwent cardiac surgery [13]. Fifty per cent of infants post cardiac surgery demonstrated delayed gross motor development at age one year [13].

Here we report the developmental outcomes of a sub-group of this cohort of children at age three years. Of particular interest was whether there was a continued disparity in development between the two groups (surgical vs healthy term-birth control), especially in relation to gross motor development. In addition we aimed to investigate the habitual physical activity and sedentary behaviour of this preschool-age group and to ascertain whether potentially delayed gross motor development was associated with decreased activity and/or increased sedentary behaviour.

## Methods

The study methods have been previously reported in detail [13]. Briefly, infants were sequentially recruited from each of the three paediatric tertiary hospitals in NSW, Australia between August 1 2006 and December 31 2008. Infants of any gestational age, who required surgery in their first 90 days, were eligible for enrolment. Healthy control infants were recruited from co-located tertiary maternity units. Ethics approval for the study was obtained from each heath service agency and parental/guardian informed consent was also given prior to study entry.

Infants enrolled in the surgical group included those who underwent open or closed cardiac surgery, or non-cardiac major surgery that required opening of a truncal body cavity (e.g. thoracotomy or laparotomy). Infants who required concurrent neurosurgery were excluded as this type of procedure is more likely to be associated with increased incidence of delayed or abnormal infant development. Infants of families who resided overseas (due to difficulty of follow-up) and/or whose parents lacked adequate English written and verbal proficiency to complete the questionnaires, were also excluded. While infants who were born premature and/or who had a known chromosomal anomaly (e.g. Down syndrome) were enrolled in the study, their results were excluded from analyses reported here as these underlying conditions are associated with developmental delay and therefore would create potential confounding factors for the current investigation.

Children were assessed at age three years using the Bayley Scales of Infant and Toddler Development, Third Edition (BSID-III) [14]. The BSID-III consists of five subscale domains: Cognition, Expressive Language, Receptive Language, Fine Motor and Gross Motor. The five subscale scores were assessed so as to identify specific differences in language and motor areas that are not identified in composite scores. The children were also assessed using this tool at age one year, enabling comparison in developmental status in all subscale areas over time for study participants. On both occasions the BSID-III was administered by one of two developmental clinicians who had undertaken BSID-III training prior to the commencement of data collection.

The BSID-III is widely used internationally to assess developmental status in infants and young children. It has been validated in the United States and is used in all the neonatal follow-up clinics in New South Wales, Australia. Some have queried whether it may underestimate developmental delay in Australian infants who are ‘at risk’ of developmental problems [15] and others have queried its validity for Australian infants or its predictive value in regards to later motor development [16, 17]. However, despite these potential issues its use in monitoring of developmental status is still supported [18]. Further, this study was a comparative study between two groups rather than a predictive study. Any potential sociocultural bias within the tool was felt to be equivalent for both the surgical and healthy peer cohort groups, enabling confidence in comparison of results between these two groups.

Habitual level of physical activity and sedentary behaviour was assessed using the Preschool-Age Physical Activity Questionnaire (Pre-PAQ). Pre-PAQ is a proxy (parental)-report questionnaire in which parents report the time their child spent in different types of physical and sedentary activities over a 3-day period (one week day and the weekend) in the home environment. These activities range from the child being stationary (Pre-PAQ Levels 1–2), moving slowly (Pre-PAQ Level 3), moving at a moderate pace (Pre-PAQ Level 4) and moving at a fast pace (Pre-PAQ Level 5). Time spent watching television or DVDs, or using other small screen devices (e.g. iPad™) is specifically reported. This questionnaire has been demonstrated to have adequate reliability and validity as a population measure of activity in preschool-age children [19]. Only a sub-sample of children was assessed using Pre-PAQ due to the time delay between commencement of the 3-year age phase of data collection (in 2009) and the publication of Pre-PAQ (in 2010). Data were collected from this sub-sample from June 2010 to August 2011. Data from the cardiac and non-cardiac surgical groups were combined to provide adequate power for data analysis.

### Data analysis

The five subscale domains of the BSID-III were analysed separately for the whole cohort. Individual scores from surgical group and healthy control peer cohort were compared against standardised norms of the BSID-III. Delay was defined as a negative deviation from normative data. The BSID-III is age-normed and has a mean of 10 and a standard deviation (SD) of 3. Mild delay was defined as a SD between > −2 to −1 SD below the mean. Moderate delay was defined as a SD > −3 to −2 SD, and severe delay as ≤ 3 SD below the mean respectively.

Data were analysed using Statistical Package for the Social Sciences for Windows (SPSS) (Version 21 SPSS Inc. Chicago, Illinois). Tests of normality were performed on all variables in accordance with procedure advocated by Peat and Barton [20] (p43), including Q-Q plot and Kolmogorov-Smirnov Test. Difference between the surgical and control group means of each subscale domain of the BSID-III were assessed using 2-sample independent *t*-tests. Level of significance was set at 0.05.

Physical activity and sedentary data from Pre-PAQ were grouped to ascertain the 3-day mean of the time each child spent (a) being active (Pre-PAQ Levels 3–5) and (b) watching/using small screen recreation devices (i.e. TV/DVD, using computers or other small screen devices). Similar to analysis of BSID-III subscale domain data, difference between group means in (1) level of activity (mins per day) and (2) time spent watching TV/DVD or other small screen devices (mins per day) was assessed using 2-sample independent *t*-tests with level of significance was set at 0.05.

## Results

**Fig. 1:**
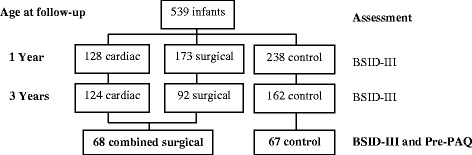
Participant numbers and outcome assessment at age 1 and age 3 years

A total of 417 term infants were assessed at age 3 years, of whom 378 had complete assessments. This represents 70 % of the cohort assessed at age 1 year. Twenty per cent of children were lost to follow-up (principally due to change of home location) and four children withdrew from the study (three for family reasons and one child was subsequently diagnosed with autism). The mixture of children lost to attrition or withdrawal was from both surgical and control groups. A sub-sample of 135 children (74 male; 61 female) was assessed for both developmental status and physical activity behaviour. There was no significant difference in mean age at time of assessment between the surgical and healthy control groups. Participant numbers and characteristics are summarised in Fig. 1 and Table 1. The characteristics of the sub-sample group were similar to those of the total group of participants assessed at age three years [12].

**Table 1:** Participant characteristics

	Surgical group	Healthy control
Number/sex	39 M: 29 F	35 M: 32 F
Mean age (SD) (months)	37 (2)	37 (1)

The BSID-III mean scores, indicative of developmental status of each group, are summarised in Table 2. A score above seven is considered as being in the average range. While both groups were within average range, children who had undergone major surgery performed significantly below their healthy peer group in the areas of cognition (*t* = −3.162, *p* = 0.002), receptive language (*t* = −3.790, *p* < 0.001) and fine motor skills (*t* = −2.153, *p* = 0.03).

**Table 2:** Mean scores on the five BSID-III subscales

Surgical group compared controls			
Variable	Surgical group mean (SD)	Control group mean (SD)	*p*-value
Cognition	9.7 (1.7)	10.7 (1.9)	0.002 **
Receptive language	10.7 (2.1)	12.0 (1.7)	<0.001 **
Expressive language	10.5 (2.3)	11.6 (2.4)	0.09 NS
Fine motor	10.7 (2.6)	11.6 (2.2)	0.03 *
Gross motor	9.5 (2.3)	10.2 (1.8)	0.06 NS

**p < 0.01, *p < 0.05, *NS* not significant

Most children at three years of age scored within the average range. However between 4–9 % of children in the surgical group demonstrated mild-moderate delay in one or more domains of cognition, expressive language, receptive language, fine motor and/or gross motor skills. No child in the healthy control peer group demonstrated any developmental delay (see Table 3).

**Table 3:** Rates of developmental delay in surgical and control groups at ages 1 year and 3 years, according to BSID-III criteria

	1 Year^a^		3 Years	
	Surgical group (*n* = 301)	Control group (*n* = 238)	Surgical group (*n* = 68)	Control group (*n* = 67)
Cognitive delay				
Mild, *n*	21	7	2	0
Moderate, *n*	2	1	1	0
Severe, *n*	0	0	0	0
Total, *n* (%)	23 (8)	8 (3)	3 (4)	0
Expressive language delay				
Mild, *n*	59	21	2	0
Moderate, *n*	16	3	2	0
Severe, *n*	3	1	0	0
Total, *n* (%)	78 (28)	25 (11)	4 (6)	0
Receptive language delay				
Mild, *n*	55	25	3	0
Moderate, *n*	2	2	1	0
Severe, *n*	2	0	0	0
Total, *n* (%)	59 (20)	27 (11)	4 (6)	0
Fine motor delay				
Mild, *n*	35	24	4	0
Moderate, *n*	2	0	1	0
Severe, *n*	1	0	0	0
Total, *n* (%)	38 (13)	24 (10)	5 (7)	0
Gross motor delay				
Mild, *n*	69	41	3	0
Moderate, *n*	42	3	3	0
Severe, *n*	19	4	0	0
Total, *n* (%)	130 (43)	48 (20)	6 (9)	0

^a^Data extracted from Walker et al. [13], p4 (cardiac and non-cardiac surgical groups combined)

Mean activity time for the surgical group was 191 mins.day^−1^, and 185 mins.day^−1^ for controls. Mean SSR time was 77 mins.day^−1^, and 83 mins.day^−1^ for the respective groups. There was no significant difference between groups for either physical activity (*p* = 0.71) or SSR time (*p* = 0.49). There was no association found between gross motor score on BSID-III and level of physical activity or SSR time.

## Discussion

The results of this study indicate that the majority of children who undergo major surgery in early infancy have satisfactory longer term developmental outcomes, despite initial delay evident in the first 12 months of life. The number of children demonstrating delay improves considerably between the ages of one and three years suggestive of “developmental catch-up” over this period.

At age one year 8–43 % of children post major surgery demonstrated developmental delay in one or more of the domains of cognition, receptive language, expressive language, fine and/or gross motor development, with delay most prevalent in gross motor development. By age 3 years only 4–9 % of children demonstrated delay in an area of their development. The domain of greatest ‘catch-up’ was in gross motor development. Forty-three percent of children who had undergone major surgery were noted to be delayed at age 12 months, whereas only 9 % children were found to be delayed at age three years.

The impact of surgery may be particularly evident on gross motor development in the first 12 months because of post-operative limitations (as a result of sternal or abdominal surgery) leading to a lack of early experience, or tolerance, of the prone position. This diminished or delayed experience could potentially delay acquisition of motor skills such as balancing in prone and crawling, and in turn delay acquisition of higher order skills of standing and walking [21]. This explanation would seem supported by our previously reported finding that infants who had undergone cardiac surgery were generally able to sit unsupported and crawl at age one year but were not yet standing and walking [13]. Of note is that these children demonstrated gross motor delay rather than movement dysfunction (i.e. abnormal movement). In other words, they displayed typical movement quality but delayed or immature gross motor skill acquisition. By age three years, children who have undergone major surgery appear to demonstrate ‘developmental recovery’.

However, while children who have undergone surgery demonstrate ‘developmental recovery’, they have not yet caught up to their peers by age 3 years. This difference was particularly evident in the areas of cognition, receptive language and fine motor development. This pattern of a generally favourable outcome but some persisting delays at age 2–3 years has also been noted in other longer-term follow-up studies of specific surgical groups including infants who survive congenital diaphragmatic hernia [22], and those who underwent cardiac surgery in their infancy [9]. Our study suggests that this pattern of developmental lag in comparison with healthy term peers exists for infants who have also undergone major surgery for other types of conditions, including infantile hypertrophic pyloric stenosis, intestinal atresia, tracheoesophageal fistula and abdominal wall defects. A similar pattern of developmental lag in comparison to term peers, at preschool age, in areas such as cognition, language and motor development has also been noted in ‘healthy’ preterm survivors (i.e. preterm children without disability such as cerebral palsy or intellectual disability) [4, 23–27].

The reasons for potential subtle vulnerability in neurodevelopment, across the range of underlying congenital anomalies noted above, have not yet been identified. However, in preterm survivors without major brain lesions, a reduction in corticomotor excitability has been noted and felt to be associated with poorer motor control in relation to fine motor skills [25]. Other influences such as adiposity, sex and socio-economic factors regarding the child’s home environment were also found to influence development in these ‘healthy’ preterm survivors [25]. Similar mechanisms/influences may also exist for children born with congenital anomalies requiring major surgery in infancy.

Cognitive capacity, language proficiency and fine motor (manual) dexterity are very relevant to school academic performance (e.g. communication, learning, and writing or keyboard skills) and socialisation. However it has yet to be determined whether the differences in abilities in these areas noted at age three years (surgical vs control) will have an impact on later comparative school performance, as has been noted in preterm infants [1, 24, 28]. Ongoing monitoring appears warranted to establish potential longer term outcomes post major surgery in infancy.

A history of major surgery did not appear to influence physical activity or sedentary behaviour (i.e. TV/DVD viewing or other small screen recreation). In this study both the surgical and healthy control peer groups met the recommended level of three hours of activity per day. However, both groups exceeded the recommended limit of 60 min of small screen recreation and engaged in an extra 16 or 22 min small screen recreation, respectively, per day. Limiting small screen recreation should be encouraged in order to meet national health recommendations. Developing positive behaviours early is important as sedentary behaviour increases with age and is independently associated with poorer health outcomes such as obesity [29, 30], type 2 diabetes [31] and cardiovascular disease [32]. Health professionals are in a position to promote healthy lifestyle habits from early infancy and childhood though anticipatory guidance management [33].

Some limitations of this phase of the study were the lower retention rate at age three years (70 %) compared with age one year (90 %). However, while the participant numbers were lower at age three years, the retention rate is comparable to similar longitudinal studies [9, 22, 34, 35]. Further, the demographic and developmental characteristics of those who remained in the study (at age three years) and those who were lost to follow-up were similar at age one year [12, 13]. Another limitation was that only a subsample of children (135 children) was assessed for both their developmental status and level of physical activity, although the demographic characteristics of the subsample were similar to the larger participant group. A strength of this longitudinal study has been the recruitment of a healthy birth cohort at the commencement of the study. The retention rate pattern for both the surgical and healthy birth cohort groups has been similar strengthening confidence in the study findings despite the loss of participants between age one and three years.

## Conclusion

In conclusion, developmental outcomes for children who undergo major surgery in early infancy are generally satisfactory. Most children who have undergone surgery are developmentally normal by age three years but they have not yet caught up to their healthy term peers, particularly in the areas of cognition, receptive language and fine motor development. This pattern of mild delay in comparison to healthy term peers, at preschool age, is similar to that seen in ‘healthy’ preterm survivors. As cognition, language and fine motor skills are very relevant to school academic performance and peer socialisation then ongoing monitoring of children, who underwent major surgery in early infancy, is warranted to determine whether the differences in developmental skills may impact upon later school performance and social behaviour as has been noted in older preterm survivors.

Physical activity and sedentary behaviour appears to be independent of developmental status. At three years children who had a history of major surgery were similarly active to their healthy control peer group and both groups were meeting national health recommendations. However, both groups exceeded recommended levels of small screen recreation. The impact of increased sedentary behaviour has yet to be determined but health professionals who deal with these families are in a position to promote positive health behaviours from early childhood and should be encouraged to do so.

## Abbreviations

BSID-III, Bayley scales of infant and toddler development, third edition; Pre-PAQ, preschool-age physical activity questionnaire; SSR, small screen recreation
